# Relationships between cardiorespiratory fitness, hippocampal volume, and episodic memory in a population at risk for Alzheimer's disease

**DOI:** 10.1002/brb3.625

**Published:** 2017-02-17

**Authors:** Ryan J. Dougherty, Stephanie A. Schultz, Elizabeth A. Boots, Laura D. Ellingson, Jacob D. Meyer, Stephanie Van Riper, Aaron J. Stegner, Dorothy F. Edwards, Jennifer M. Oh, Jean Einerson, Claudia E. Korcarz, Rebecca L. Koscik, Maritza N. Dowling, Catherine L. Gallagher, Cynthia M. Carlsson, Howard A. Rowley, Barbara B. Bendlin, Sanjay Asthana, Bruce P. Hermann, Mark A. Sager, James H. Stein, Sterling C. Johnson, Ozioma C. Okonkwo, Dane B. Cook

**Affiliations:** ^1^ William S. Middleton Memorial Veterans HospitalMadisonWIUSA; ^2^ Department of KinesiologyUniversity of Wisconsin School of EducationMadisonWIUSA; ^3^ Geriatric Research Education and Clinical CenterWilliam S. Middleton Memorial Veterans HospitalMadisonWIUSA; ^4^ Wisconsin Alzheimer's Disease Research CenterUniversity of Wisconsin School of Medicine and Public HealthMadisonWIUSA; ^5^ Wisconsin Alzheimer's InstituteUniversity of Wisconsin School of Medicine and Public HealthMadisonWIUSA; ^6^ Department of KinesiologyIowa State University College of Human SciencesAmesIAUSA; ^7^ Department of Family Medicine and Community HealthUniversity of WisconsinMadisonWIUSA; ^8^ Division of CardiologyUniversity of Wisconsin School of Medicine and Public HealthMadisonWIUSA; ^9^ Department of Biostatistics & Medical InformaticsUniversity of Wisconsin School of Medicine and Public HealthMadisonWIUSA; ^10^ Department of NeurologyUniversity of Wisconsin School of Medicine and Public HealthMadisonWIUSA

**Keywords:** APOE‐e4, cognition, exercise, family history, physical activity

## Abstract

**Introduction:**

Cardiorespiratory fitness (CRF) has been shown to be related to brain health in older adults. In individuals at risk for developing Alzheimer's disease (AD), CRF may be a modifiable risk factor that could attenuate anticipated declines in brain volume and episodic memory. The objective of this study was to determine the association between CRF and both hippocampal volume and episodic memory in a cohort of cognitively healthy older adults with familial and/or genetic risk for Alzheimer's disease (AD).

**Methods:**

Eighty‐six enrollees from the Wisconsin Registry for Alzheimer's Prevention participated in this study. Participants performed a graded maximal exercise test, underwent a T‐1 anatomical magnetic resonance imaging scan, and completed the Rey Auditory Verbal Learning Test (RAVLT).

**Results:**

There were no significant relationships between CRF and HV or RAVLT memory scores for the entire sample. When the sample was explored on the basis of gender, CRF was significantly associated with hippocampal volume for women. For men, significant positive associations were observed between CRF and RAVLT memory scores.

**Summary:**

These results suggest that CRF may be protective against both hippocampal volume and episodic memory decline in older adults at risk for AD, but that the relationships may be gender specific.

## 1 Introduction

Alzheimer's disease (AD) currently affects over 5 million Americans, with the incidence projected to climb to 13.5 million by the year 2050 (Barnes & Yaffe, 2011). Individuals with a family history of AD or a genetic predisposition have the greatest likelihood of developing the disease (Duijn et al., 1991; Gatz et al., 2006). There is a critical need to discover treatments to delay disease progression in individuals that are at risk for AD.

The hippocampus plays a crucial role in cognitive processing associated with memory function (Tulving & Markowitsch, 1998), and accelerated atrophy of this region is an early feature of AD (Jack et al., 2000). Of particular interest is the relationship between hippocampal volume and episodic memory (Kramer et al., 2005; Mormino et al., 2009; Stoub, Rogalski, Leurgans, Bennett, & deToledo‐Morrell, 2010). Episodic memory is one of the initial cognitive domains to decline with age (Nilsson, 2003; Schaie, 1994) and is dependent on the hippocampus for both memory formation and consolidation (Henson, 2005; Kirwan & Stark, 2004; Tulving & Markowitsch, 1998). These data suggest that preserving hippocampal volume may be critical for maintaining cognitive health and potentially delaying AD in older adulthood. However, to date, the relationship between hippocampal volume and episodic memory is equivocal (Chen, Chuah, Sim, & Chee, 2010; Van Petten, 2004).

Available evidence suggests that exercise may benefit both the hippocampus and memory. In animals, exercise enhances hippocampal neurogenesis and memory performance (Nichol, Deeny, Seif, Camaclang, & Cotman, 2009; Olson, Eadie, Ernst, & Christie, 2006; van Praag, Kempermann, & Gage, 1999; van Praag, Shubert, Zhao, & Gage, 2005). Similar results have been reported in human studies, with 12 months of structured exercise increasing hippocampal volume in older, cognitively healthy, sedentary populations (Erickson et al., 2011; Niemann, Godde, & Voelcker‐Rehage, 2014). Conversely, cessation from exercise training has been shown to result in decreased blood flow to the hippocampus for master athletes (Alfini et al., 2016). Although this research has demonstrated neurobiological adaptations to chronic exercise training, it is unclear whether changes in cardiorespiratory fitness (CRF) are necessary for these adaptations to manifest.

The relationships between CRF, hippocampal volume, and memory in cognitively healthy older adults without known risk factors for AD are currently unclear. For CRF, the data are equivocal with some studies reporting significant positive associations between CRF and hippocampal volume (Bugg, Shah, Villareal, & Head, 2012; Erickson et al., 2009), and others not observing such associations (Honea et al., 2009; Niemann et al., 2014; Vidoni, Honea, Billinger, Swerdlow, & Burns, 2012). Differing results may be in part due to the method of obtaining hippocampal volume (e.g., manual tracing, automated segmentation), or the inclusion criteria for a valid VO_2peak_ test. Thus, it is important that standardized criteria be employed and adhered to when determining relationships between brain volume and aerobic fitness.

Similarly, the relationship between CRF and episodic memory in older adults also appears to be equivocal. In older adults, CRF has been reported to be positively associated episodic memory (Etnier et al., 2007; Hayes, Forman, & Verfaellie, 2016; Zhu et al., 2014), while others have not observed such associations in similar populations (Bugg et al., 2012; Maass et al., 2015). Moreover, both self‐reported physical activity (Pizzie et al., 2014) and estimated fitness (Boots et al., 2014; Freudenberger et al., 2016) have been shown to be positively associated with episodic memory in older adulthood.

It is possible that hippocampal volume may mediate the relationship between CRF and memory performance (Erickson et al., 2009); enhanced CRF may cause an increase in hippocampal volume, which then leads to enhanced memory performance (Erickson et al., 2011). However, here too, the evidence is contradictory. Varma, Chuang, Harris, Tan, and Carlson, (2015) reported a positive association between physical activity and hippocampal volume, but no significant association between physical activity and episodic memory. ten Brinke et al. (2015) reported a negative relationship between hippocampal volume and episodic memory performance following an exercise training intervention.

A clearer picture may emerge by focusing research on older adults with known risk factors for AD, thus reducing some of the heterogeneity that is inherent in elderly populations. Recent evidence suggests that self‐reported physical activity is positively associated with both hippocampal volume and memory in men and women at risk for AD (Boots et al., 2014; Okonkwo et al., 2014; Smith, Nielson, Woodard, Seidenberg, & Rao, 2013, 2014). However, whether these findings extend to CRF is unknown. The purpose of this study was to determine the relationship between objectively measured CRF, hippocampal volume, and episodic memory in a cohort of cognitively healthy men and women with familial and/or genetic risk for AD. Based on data suggesting gender differences between exercise and both hippocampal volume (Varma et al., 2015) and cognition (Colcombe & Kramer, 2003; Middleton, Kirkland, & Rockwood, 2008) that may be explained by altered metabolic effects of exercise (Baker et al., 2010), we also explored potential gender differences within our sample. We hypothesized that CRF would be positively associated with both hippocampal volume and episodic memory. To specifically test the influence of CRF, we employed standardized criteria for the determination of aerobic capacity and only included participants who met these *a priori* set criteria.

## 2 Materials and Methods

### 2.1 Participants

All participants were recruited from the Wisconsin Registry for Alzheimer's Prevention (WRAP). The WRAP is a longitudinal registry composed of more than 1,500 cognitively healthy adults and is enriched for risk factors of AD (Sager, Hermann, & Rue, 2005). WRAP participants are free of major medical conditions (e.g., neurological diseases and psychiatric disorders), and have been deemed safe for neuroimaging procedures as participants undergo waves of testing including neuropsychological evaluations and neuroimaging protocols. For this study, WRAP participants were recruited to participate in a cardiorespiratory fitness test via phone calls and recruitment letters. Estimates for adequate sample size were calculated using G*Power 3.1 (Faul, Erdfelder, Buchner, & Lang, 2009). Assuming a relatively small effect size (*f* ^2^ = 0.15; approximately 10% of variance accounted for), level of significance (alpha)  = .05, power (1‐beta)  = .8, and six predictors for a two‐tailed test, the necessary sample size was estimated to be fifty‐five. Eighty‐six older adults (ages 50–75) were screened, deemed eligible, and agreed to participate in this study. Selection was based on risk for AD and availability of neuropsychological and neuroimaging data. All participants had either a parental family history (FH) of AD or were apolipoprotein epsilon 4 allele (APOE‐e4) positive, indicating increased genetic risk for developing AD. Study participants were determined to be cognitively healthy using the Mini‐Mental state examination (MMSE ≥ 24) and were excluded from fitness testing for any of the following: documented vascular disease, type 1 or 11 diabetes mellitus, severe untreated hypertension (>200/100 mmHg), and the inability to safely walk on a treadmill. Participants were not enrolled in any exercise trials at the time of testing. The University of Wisconsin Institutional Review Board approved all study procedures, and informed consent was obtained from all individual participants.

### 2.2 Cardiorespiratory fitness assessment

Exercise tests were conducted by a certified exercise physiologist along with a trained exercise specialist following a resting twelve‐lead electrocardiogram (ECG) assessment. The exercise test was conducted on a motor‐driven treadmill using a modified Balke protocol (Balke & Ware, 1959). Comfortable walking speeds were determined during a warm‐up period and kept constant for the duration of testing. The majority of participants walked at 3.5 miles per hour. However, if the participant indicated that this speed was uncomfortable, a slower walking speed was chosen. Following a two‐minute warm‐up at 0% grade, the grade of the treadmill was increased by 2.5% every two minutes until the participant reached volitional exhaustion or indicated they could no longer continue. During the exercise test, oxygen consumption (VO_2_), carbon dioxide production (VCO_2_), minute ventilation (VE), respiratory exchange ratio (RER), and work rate were obtained using a metabolic cart (TrueOne^®^ 2400 Parvomedics, Sandy, UT, USA) and a two‐way non‐rebreathing mask. Heart rate (HR) was continuously measured through a twelve‐channel ECG device (Schiller CS‐200 Exercise Stress System, Baar, Switzerland). The system was calibrated using standard procedures within four hours of the exercise test to ensure accuracy.

Peak effort was determined based on American College of Sports Medicine (ACSM) criteria of meeting at least two of the following: (1) RER ≥ 1.1, (2) change in VO_2_ < 200 ml with an increase in work, (3) rating of perceived exertion (RPE) of 17 or greater (Borg, 1982), and (4) achieving at least 90% of age‐predicted maximal heart rate (220‐age) (Lippincott, Williams, & Wilkins, 2013). CRF was defined as the highest oxygen consumption value recorded at peak effort (VO_2_peak, ml kg^−1^ min^−1^) during the exercise test when the criteria were met. The ACSM criteria were chosen *a priori* and strictly adhered to, and those not satisfying criteria were not included in subsequent data analyses. These methods of exclusion were implemented to avoid potential age‐driven relationships when volitional effort was not achieved (e.g., the oldest participants stopping early).

### 2.3 Neuroimaging protocol

**Figure 1:**
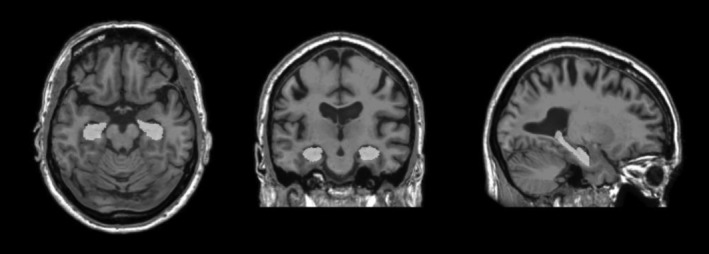
Image of the hippocampus in the axial, coronal, and sagittal view

Magnetic resonance images (MRI) were acquired on a GE X750 Discovery 3.0T scanner with an eight‐channel phased‐array head coil (General Electric, Waukesha, WI, USA). Three‐dimensional T1‐weighted inversion recovery‐prepared SPGR anatomical sequences were collected using the following parameters: TI/TE/TR = 450 ms/3.2 ms/8.2 ms, flip angle = 12°, slice thickness = 1 mm no gap, FOV = 256, matrix size = 256 × 256, yielding a voxel resolution of 1 mm × 1 mm × 1 mm. Hippocampal volume was derived from the images using the Freesurfer image analysis suite version 5.1.0 (http://surfer.nmr.mgh.harvard.edu/) with automated volumetric segmentation (Fischl, 2012). Further technical processing details are described in previous publications (Dale, Fischl, & Sereno, 1999; Fischl, Sereno, & Dale, 1999). Freesurfer segmentation methods are widely used and have been validated against gold‐standard manual segmentation methods (Mulder et al., 2014; Tae, Kim, Lee, Nam, & Kim, 2008). All images were visually inspected slice by slice to ensure they were accurately reconstructed and without topological defects and edited by trained personnel if needed (Figure 1). On average, scans were conducted within 10.2 ± 6.3 months of the exercise test. All hippocampal volumes are expressed as a percentage of intracranial volume (ICV) to account for differences in overall head size.

### 2.4 Episodic memory assessment

The Rey Auditory Verbal Learning Test (Schmidt, 1996) is a common neuropsychological tool used to evaluate episodic memory (Sager et al., 2005). It has been shown to be sensitive to cognitive impairment and predicts AD years prior to diagnosis (Rabin et al., 2009; Tierney, Yao, Kiss, & McDowell, 2005). Further, research has demonstrated an association between RAVLT performance and hippocampal volume in cognitively healthy and early‐stage AD participants (Petersen et al., 2000). For this study, four separate episodic memory scores including total learning, delayed recall, recognition recall, and an overall composite score. Total learning was the sum of correctly identified words over trials I‐V. Delayed recall score was the sum of correctly identified words in trial VII. Recognition recall score was the sum of correctly identified words from list A when presented with a list of 50 words. The composite score is the summed average of all three RAVLT episodic memory scores, and thus served as our primary episodic memory variable of interest (Lezak, 2004). On average, the RAVLT was conducted 9.48 ± 6.6 months from the exercise test and 5.4 ± 7.8 months from the MRI. Additional RAVLT metrics were explored and are reported in the supplemental data section.

### 2.5 Statistical analyses

Independent‐samples *t* tests were used to compare demographics, and analyses of covariance (ANCOVA) were conducted to compare CRF, episodic memory, and hippocampal volume differences between men and women. Separate multiple linear regression analyses were used to assess the relationship between CRF and hippocampal volume and between CRF and episodic memory with the significance level (α) set at .05. Within these models, variables with known associations to either the predictor (CRF) or outcome (hippocampal volume and episodic memory score) variables were chosen *a priori* and added as covariates to the regression model. These variables included age, gender, body mass index (BMI), and education. On average, participants had undergone both the MRI scan and episodic memory test within 10 months of the exercise test. However, because the time intervals varied among participants, this was also controlled for in our analyses. Unless indicated otherwise, all reported regression models included the variables age, gender, BMI, years of education, and time interval between CRF test and MRI/RAVLT. Secondary analyses were used to explore the effect of gender. All regression analyses were conducted using IBM SPSS, version 22.0.

## 3 Results

### 3.1 Sample

Eighty‐six cognitively healthy (MMSE = 29.3 ± 0.95) participants (mean age = 63.6 ± 5.9 years) completed the study. Seven participants possessed the APOE‐e4 allele but had no FH, 40 participants had a FH of AD and were APOE‐e4 negative, and 39 participants were both FH positive and APOE‐e4 positive. Additional participant demographics are presented in Table 1. Nineteen participants (22.1% of the sample) were excluded from analyses, as their exercise tests did not satisfy the criteria for a VO_2_peak test. The exercise physiologist supervising the exercise stopped five of these tests due to safety concerns (arrhythmia, dyspnea, hypertension), one participant stopped early due to mask discomfort, and 13 participants (15.1% of the sample) stopped the test prior to attaining peak criteria due to fatigue. The remaining 67 participants met standardized criteria for peak effort. Participants not satisfying CRF inclusion criteria recorded lower maximal HR, RER, and RPE at peak effort than participants satisfying criteria (*p *<* *.05; Table 2). Participants excluded for not satisfying CRF criteria (*n *= 19) were older, and had poorer episodic memory than those satisfying criteria (*n *= 65) (*p *<* *.05; Table 1); however, hippocampal volumes were not significantly different (*p *=* *.06; Table 1). The groups did not differ on any other measured demographic characteristics. Of the participants satisfying the exercise test criteria, 10 were excluded as their MRI data were not available for analysis and two were excluded for not having completed the RAVLT. Thus, the analytic sample consisted of 57 participants (mean age = 62.6 ± 6 years) for the CRF and hippocampal volume analysis, and 65 participants (mean age = 62.9 ± 6 years) for the CRF and episodic memory analysis.

**Table 1:** Participant demographics stratified by gender and analyses

Variable	Total sample	Women	Men	CRF‐HV analysis	CRF‐EM analysis	Excluded participants
Sample size	86	53	33	57	65	19
Female, %	61.6	100	0	61.4	61.5	63
Age, years	63.6 (5.9)	63.5 (6.1)	63.6 (5.5)	62.6 (6.0)	62.9 (6.0)	66.2 (4.5)^a^
MMSE, score	29.3 (0.95)	29.3 (0.83)	29.2 (1.1)	29.4 (1.0)	29.3 (0.98)	29.1 (0.88)
BMI, kg/m^2^	28.3 (5.3)	28.8 (6.3)	27.7 (3.3)	27.6 (4.8)	27.6 (4.6)	30.6 (7.2)
Education, years	16.3 (2.4)	15.9 (2.4)	17 (2.2)	16.6 (2.1)	16.5 (2.1)	15.6 (3.0)
APOE4+ and FH −, %	8.1	3.8	15.2	8.8	7.7	10.5
FH + and APOE4 −, %	46.5	49.1	42.4	38.6	41.5	57.9
APOE4+ and FH +, %	45.3	47.2	42.4	52.6	50.8	31.6
Caucasian, %	94.2	96	91	93	93.8	95
Attain Peak Effort, %	77.9	77.4	78.8	100	100	0
VO_2_peak, ml ^−1^kg^−1^ min^−1^	25.9 (6.9)	23.2 (5.8)	30.1 (6.4)	26.8 (6.0)	26.6 (5.8)	22.9 (9.3)
HV, % ICV	0.57 (0.06)	0.58 (0.06)	0.56 (0.06)	0.58 (0.06)	0.58 (0.06)	0.55 (0.08)
EM, composite score	34.3 (4.9)	35.8 (4.3)	31.9 (4.8)	35 (4.4)	34.9 (4.4)	32.3 (5.9)^a^

Values indicate mean score and standard deviation, unless otherwise indicated. HV = hippocampal volume; EM = episodic memory, MMSE = Mini‐Mental state examination; BMI = body mass index; APOE4 = the epsilon 4 allele of the apolipoprotein E gene; VO_2_peak = peak oxygen consumption during exercise test.

a Significantly different than the analytic sample of included participants.

**Table 2:** Exercise data at peak stratified by criteria

Exercise variable	Included participants	Excluded participants	Cohen's *d* effect size
Sample size	67	19	–
HR max, %	101.58 (7.7)^a^	94.32 (10.8)	0.8
RER, value	1.102 (0.082)^a^	0.996 (0.065)	1.4
RPE, value	17.67 (1.51)^a^	15.58 (1.84)	1.2
VO_2_peak, ml kg^−1^ min^−1^	26.68 (5.89)	22.85 (9.31)	0.5

a Values for included participants were significantly higher (*p *<* *.05) compared to excluded participants.

### 3.2 Gender differences

Men in this sample were taller, heavier, and possessed greater CRF (*p *<* *.05). Women performed significantly better on the RAVLT episodic memory test than men (*p *<* *.05) with medium‐to‐large effect sizes observed (*d = *0.7–1.0; Table 3). Women also exhibited superior performance compared to two separate assessments of age‐matched normative values (Normative values: Total recall: 47.2, 43.4. Delayed recall: 9.4, 8.8. Recognition recall:12.8, 13.5; refer to Mitrushina, Boone, Razani, & D'Elia, 2005; Strauss, Sherman, & Spreen, 2006) on the RAVLT (*p *<* *.05).

**Table 3:** RAVLT performance stratified by gender

Variable	Men	Women	Cohen's *d* effect size
Sample size	25	40	–
Total Recall	47.2 (7.2)^a^	54.1 (6.3)	1.0
Delayed Recall	9.4 (2.3)^a^	11.3 (2.5)	0.8
Recognition Recall	13.5 (1.5)^a^	14.3 (0.9)	0.7
Composite Score	32.4 (4.2)^a^	36.5 (3.8)	1.0

a Scores for male participants were significantly lower (*p *<* *.05) compared to women participants when controlling for age, education, and BMI.

### 3.3 CRF and hippocampal volume

**Figure 2:**
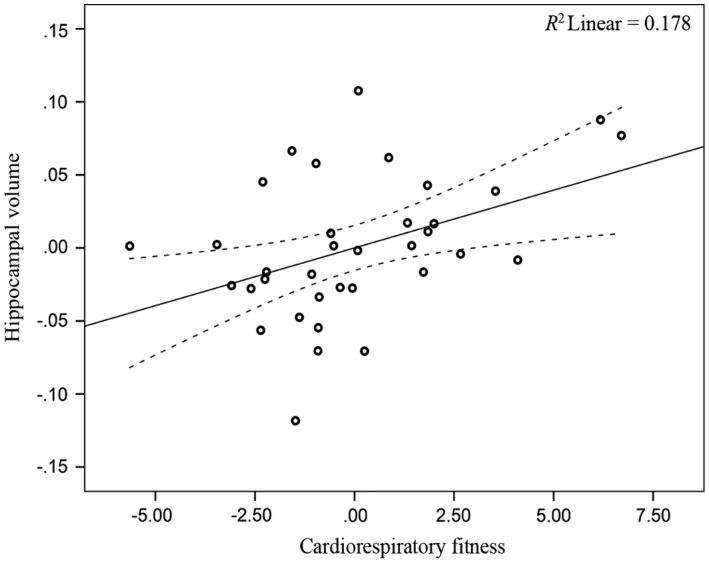
Relationship between CRF and hippocampal volume (women). Data are partial regression plots accounting for the covariates included in the linear regression model including age, BMI, education, and time interval. The individual regression slope is indicated with the solid line. The 95% confidence interval is indicated with the dotted line

**Figure 3:**
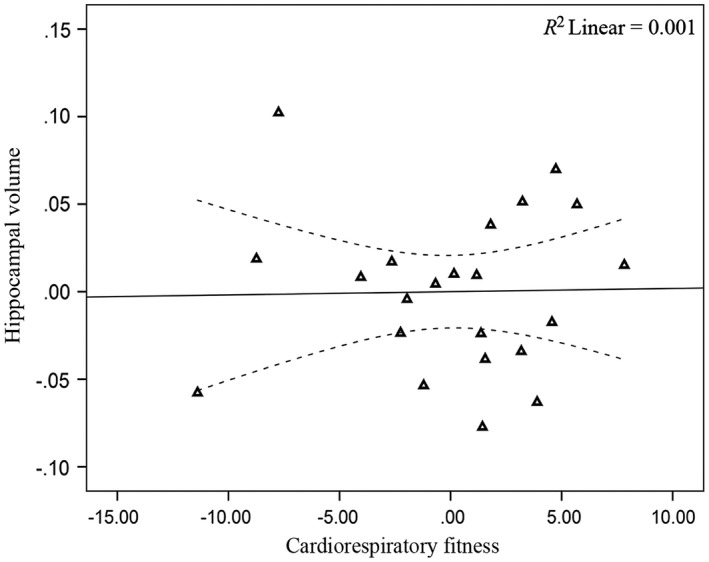
Relationship between CRF and hippocampal volume (men)

Across the full analytic sample (*n *= 57), CRF was not significantly associated with hippocampal volume (*p* = .248; Table 4). When the analyses were stratified by gender (women *n *= 35, men *n *= 22), CRF was significantly associated with hippocampal volume for women (*p *=* *.018; Table 4; Figure 2), but not men (*p *=* *.938; Table 4; Figure 3). Adding a CRF × Gender interaction term to further test the observed gender differences did not result in a significant interaction (*p *= .177). Due to the observed gender‐specific association, we examined bivariate correlations within the regression models to determine whether relationships between the covariates and hippocampal volume were also gender specific. Results demonstrated that age and years of education were negatively associated with hippocampal volume for women (*r *=* *−.53, −.49; *p *<* *.05), but not men (*r *=* *−.24, .19; *p *>* *.05). No other correlations were statistically significant.

**Table 4:** Multiple linear regression for predicting hippocampal volume

Independent variables	R^2^			B			SE B			β			*p*‐value		
	ES	M	W	ES	M	W	ES	M	W	ES	M	W	ES	M	W
CRF				.002	.000	.008	.002	.002	.003	.234	.022	.667	.248	.938	.018
Age				−.003	−.001	.000	.001	.002	.002	−.332	−.176	−.033	.034	.560	.876
Gender				.018	–	–	.018	–	–	.157	–	–	.311	–	–
BMI				.001	.001	.004	.002	.004	.002	.106	.086	.391	.497	.774	.062
Education				−.006	.004	−.014	.003	.006	.005	−.228	.179	−.454	.070	.549	.004
Time Interval				.003	.009	.007	.014	.027	.015	.032	.083	.068	.800	.750	.621
Model predicting HV	.266	.082	.506										.013	.913	.001

ES = entire sample; M = men; W = women. *R* ^2^: denotes the proportion of variance explained by the model. *B*: denotes the variable estimate. *SE B*: denotes the standard error of the variable estimate. β: denotes the standardized estimate. Sample Sizes: ES = 57, W = 35, M = 22.

### 3.4 CRF and episodic memory

**Figure 4:**
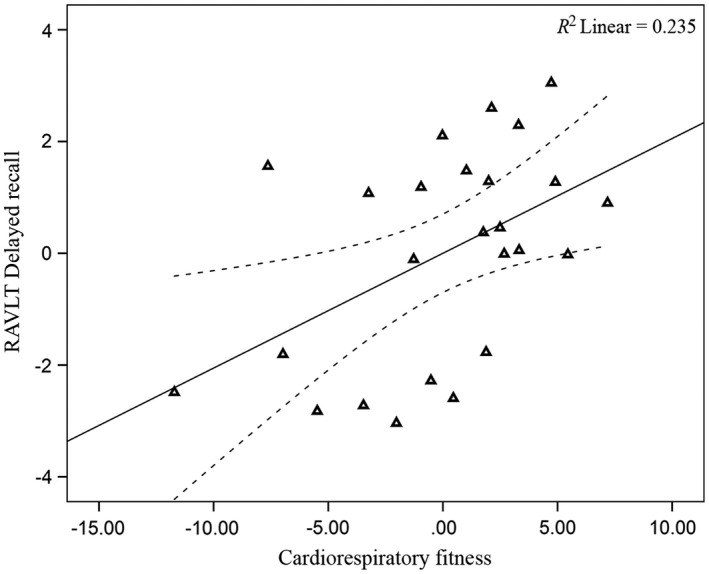
Relationship between CRF and RAVLT delayed recall (men). Data are partial regression plots accounting for the covariates included in the linear regression model including age, BMI, education, and time interval. The individual regression slope is indicated with the solid line. The 95% confidence interval is indicated with the dotted line

**Figure 5:**
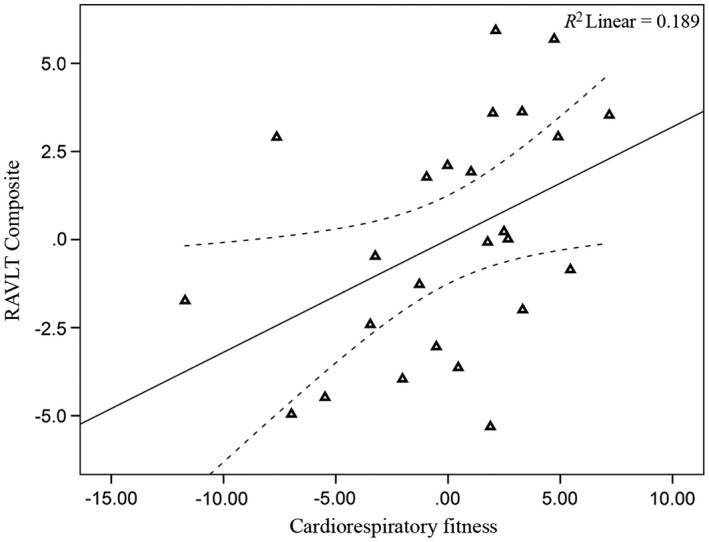
Relationship between CRF and RAVLT composite (men). (See Figure 4 legend for explanation)

**Figure 6:**
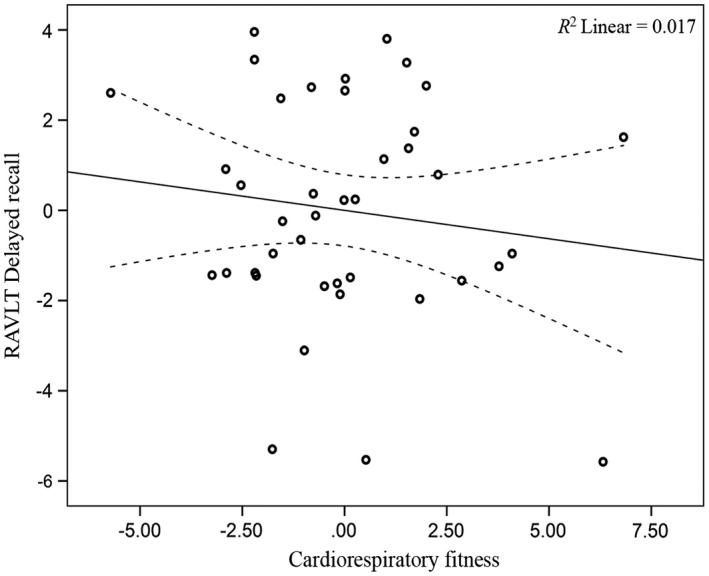
Relationship between CRF and RAVLT delayed recall (women) (See Figure 4 legend for explanation)

**Figure 7:**
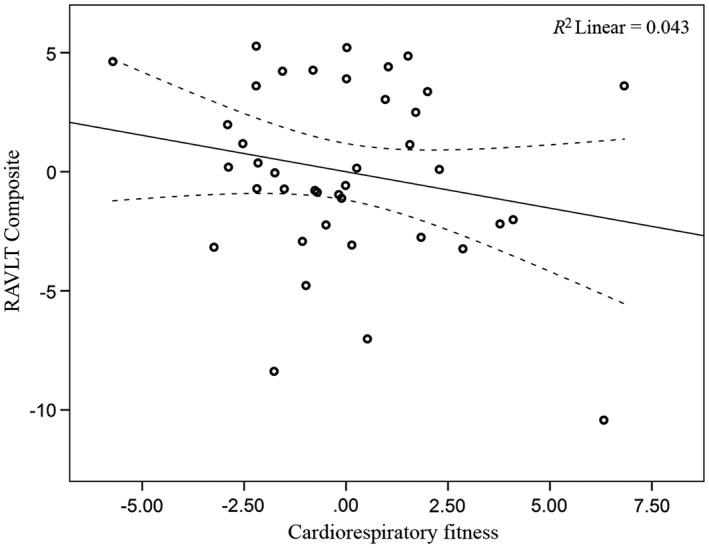
Relationship between CRF and RAVLT composite (women). (See Figure 4 legend for explanation)

Across the full analytic sample (*n *= 65), there were no significant associations between CRF and RAVLT episodic memory scores (*p *>* *.05; Table 5). However, when examining results separately by gender, CRF was significantly associated with RAVLT delayed recall score (*p *=* *.026; Figure 4) and the composite memory score (*p *=* *.049; Table 5; Figure 5) for men (*n *= 25). No significant associations between CRF and RAVLT episodic memory scores were observed for women (*n *= 40, *p *>* *.05; Table 5; Figures 6 and 7). The CRF × Gender interaction was not significant for the RAVLT delayed (*p *= .303) or RAVLT composite score (*p *= .251) in either of the regression models. Further, there were no significant associations between hippocampal volume and RAVLT episodic memory scores for either the entire sample or separately for men and women (*p *>* *.05).

**Table 5:** Multiple linear regression for predicting episodic memory (composite)

Independent variables	R^2^			B			SE B			β			*p*‐value		
	ES	M	W	ES	M	W	ES	M	W	ES	M	W	ES	M	W
CRF				.092	.320	−.306	.142	.152	.248	.120	.413	−.412	.519	.049	.227
Age				−.112	−.093	−.213	.104	.168	.163	−.153	−.129	−.345	.289	.587	.201
Gender				4.46	–	–	1.29	–	–	.494	–	–	.001	–	–
BMI				.100	.719	−.224	.134	.284	.179	.104	.534	−.314	.461	.020	.220
Education				.159	.342	.173	.232	.368	.337	.077	.190	.091	.494	.365	.611
Time Interval				1.81	2.49	1.08	.910	1.48	1.14	.226	.327	.158	.052	.109	.350
Model predicting EM	.291	.505	.081										.002	.014	.700

ES = entire sample; M = men; W = women. *R* ^2^: denotes the proportion of variance explained by the model. *B*: denotes the variable estimate. *SE B*: denotes the standard error of the variable estimate. β: denotes the standardized estimate. Sample Sizes: ES = 65, W = 40, M = 25.

## 4 Discussion

In this cohort of cognitively healthy but at‐risk adults, we did not observe significant positive associations between fitness and either hippocampal volume or episodic memory. However, we did observe significant relationships suggestive of gender‐specific effects. In women, we observed a significant positive association between CRF and hippocampal volume; for men, there were significant positive associations between CRF and episodic memory. These results suggest that CRF may protect against hippocampal atrophy in asymptomatic women at elevated risk for AD, although CRF was not associated with a memory benefit. Further, for men, CRF was not associated with hippocampal volume but still was associated with improved memory. Because we did not observe significant gender by fitness interactions for either hippocampal volume or episodic memory, the gender specificity of our findings are uncertain. Thus, the discussion of the observed results will be descriptive and should be considered with respect to this limitation.

Overall, the hypotheses that CRF would be significantly and positively associated with hippocampal volume and episodic memory performance were not supported. We based the premise of our study on data that suggest a positive relationship between hippocampal volume and episodic memory in cognitively healthy older adults (Kramer et al., 2005; Mormino et al., 2009; Petersen et al., 2000). However, there are meta‐analytic data (Van Petten, 2004) along with large‐scale cross‐sectional data (Chen et al., 2010) that suggest weak empirical support for this relationship. Further, a recent exercise trial observed increased hippocampal volume was associated with reduced episodic memory performance on the RAVLT (ten Brinke et al., 2015). Taken together, these studies suggest that the data are equivocal. We attempted to further clarify this conflicting literature by applying strict criteria for determining aerobic fitness and testing a population with either a familial and/or genetic risk for AD.

These novel findings are of particular interest due to the at‐risk nature of the participants. Research has demonstrated a hereditary component to AD. Specifically, individuals with parental family history of AD are three times more likely to develop the disease compared to those with no familial history (Duijn et al., 1991). Further, there is a strong genetic component, with those at greatest risk being carriers of APOE‐e4 (Jack et al., 2010). If the APOE‐e4 allele is inherited, the chance of developing AD doubles (Vardarajan et al., 2014). It is estimated that 40–65% of individuals diagnosed with AD carry one or two copies of the APOE‐e4 allele (Farrer et al., 1997; Saunders et al., 1993). The findings of the current study suggest that possessing greater CRF may slow the temporal progression of the disease even in the presence of risk factors for AD, but that this protective effect may not apply equally for men and women.

### 4.1 CRF and hippocampal volume

To date, the authors are aware of five studies examining the association between CRF (VO_2_peak) and hippocampal volume in cognitively healthy older adults, the results of which have been equivocal. Erickson et al. (2009) observed significant positive associations between CRF and both left and right hippocampal volumes in cognitively healthy, sedentary older adults. Similar positive findings between CRF and hippocampal volume were reported for obese (BMI ≥ 30), cognitively healthy older adults (Bugg et al., 2012). In contrast, Niemann et al. (2014) did not find significant associations between CRF and hippocampal volume in cognitively intact older adults. Other studies have found associations in early‐stage AD participants, but not in participants who are cognitively healthy. Honea et al. (2009) reported positive associations between right parahippocampal volume and CRF in early‐stage AD participants. A two‐year follow‐up study by this same group reported significant associations between CRF change and left parahippocampal volume in the same early‐stage AD participants (Vidoni et al., 2012). Interestingly, neither baseline CRF nor change in CRF was related to hippocampal volume in the cognitively healthy participants of either study (Honea et al., 2009; Vidoni et al., 2012). More recently, change in CRF following an exercise intervention has been positively associated with hippocampal head volume (Maass et al., 2015) and hippocampal tissue density (Kleemeyer et al., 2015) in cognitively healthy older adults. Overall, current research demonstrates inconsistent associations between CRF and hippocampal volume.

There are several potential explanations for the conflicting results including the methods used to measure hippocampal volume, the criteria applied to determine CRF, and the cognitive status of the participants. One important limitation of the current literature is the use of nonstandard criteria for ascertaining CRF, likely due to the concern for an older adult's ability to produce a peak effort exercise test and/or safety concerns. While such concerns are reasonable, they may result in underestimation of peak effort, which in turn could obscure or artificially inflate relationships for outcomes that are age dependent such as brain structure and cognitive function, as these are known to decline with age (Jack et al., 2010; Morra et al., 2009; Nilsson, 2003; Pfefferbaum et al., 2013). Adherence to standardized criteria for determining CRF is important to avoid potential bias with the inclusion of invalid tests (i.e., values that are below an individual's true aerobic capacity), thus making results more difficult to interpret. This is particularly important when testing older adults, as these individuals may be less inclined to provide maximal efforts during exercise testing (Church et al., 2008). The present investigation addressed this potential limitation by adhering to standardized and widely accepted criteria for measuring CRF (VO_2_peak) and including only those who met these criteria in the statistical analyses.

Participants who did not satisfy our *a priori* criteria were older and exhibited poorer episodic memory than those satisfying criteria. However, these individuals were not necessarily the least functional. Examination of perceived exertion and metabolic data (**Table** 2) show that these individuals stopped at an average RPE of 15 (hard effort) and an average RER of 0.99, both values well below criteria. However, their heart rate data were within criteria (94% of age‐predicted peak). These data suggest that participants could have provided a greater effort and that from a metabolic perspective, their cardiorespiratory response was not the limiting factor. Under these circumstances, their recorded CRF is likely an underestimation of their true aerobic capacity, and thus an invalid measure. There are numerous reasons why individuals choose to stop during maximal exercise tests (Lippincott, Williams, & Wilkins, 2013), and the present study cannot definitively answer why certain individuals chose to stop early. Future research that systematically examines the consequences of implementing various CRF criteria for testing in older adults is needed.

For women, a significant positive association between CRF and hippocampal volume was observed. This finding is consistent with a recent study that reported positive associations between steps per day (i.e., physical activity) and hippocampal volume in women but not men (Varma et al., 2015). Other randomized control trials have also found evidence for gender‐specific effects (van Uffelen, Chinapaw, van Mechelen, & Hopman‐Rock,^2008^; Vaughan, Morris, Shum, O'Dwyer, & Polit, 2012), with data suggesting the superior cognitive response in women may be the result of gender differences in exercise‐induced hormonal glucometabolic and hypothalamic–pituitary–adrenal axis responses (Baker et al., 2010). This may explain the gender‐specific findings reported from the epidemiological Canadian Study of Health and Aging where moderate‐to‐high exercise participation resulted in reduced risk of cognitive impairment for women but not men (Middleton et al., 2008). Alternatively, the present study's results may be explained by gender differences of the current sample. As expected, a negative association between age and hippocampal volume in the entire sample was observed. However, when examining the sample split by gender, the negative association only remained for the women. Although the propensity of the literature supports similar age‐related hippocampal volume loss for men and women (Jack et al., 2015; Mu, Xie, Wen, Weng, & Shuyun, 1999; Raz et al., 2004, 2005), there are reports of both women‐ and men‐specific age–hippocampal volume relationships (Murphy et al.,^1996^; Pruessner et al., 2001). For the current sample, the lack of significant association in the men may be explained by this gender not experiencing a similar age‐related trajectory in hippocampal volume, and therefore not experiencing the benefits of CRF on hippocampal volume.

### 4.2 CRF and episodic memory

Analyses of the entire sample did not reveal significant associations between CRF and any of the four RAVLT episodic memory scores. However, there were significant associations between CRF and both RAVLT delayed recall score and RAVLT composite memory score for the men. In older adults, large‐scale cross‐sectional studies have reported contradictory associations. Positive associations between CRF/physical activity and RAVLT performance have been reported (Boots et al., 2014; Pizzie et al., 2014; Zhu et al., 2014) while others studies did not observe such associations (Maass et al., 2015; Varma et al., 2015). Randomized controlled trials provide evidence that aerobic exercise (Klusmann et al., 2010) and resistance exercise (Cassilhas et al., 2007) training can improve episodic memory in cognitively healthy older adults. Additionally, changes in physical activity/CRF following an exercise intervention have been positively associated with episodic memory performance (Maass et al., 2015; Ruscheweyh et al., 2011). The findings of the current study are in part consistent and show that in addition to self‐reported physical activity and estimated fitness, objectively measured CRF (VO_2_peak) is associated with episodic memory in at‐risk older adult men.

The lack of an association for the women in the present study was unexpected, but may be explained by their level of cognitive performance. The women performed better on all four RAVLT episodic memory measures compared to men, which supports extant literature (Gale, Baxter, Connor, Herring, & Comer, 2007; Geffen, Moar, O'hanlon, Clark, & Geffen, 1990; Van Der Elst, van Box Tel, van Breukelen, & Jolles, 2005). Further, the medium‐to‐large effect sizes observed between genders in this study closely resemble previous research (Gale et al., 2007). In addition to exhibiting superior episodic memory performance compared to men, they performed significantly better than age‐matched normative values (Mitrushina et al., 2005; Strauss et al., 2006). Because our sample was cognitively intact and not showing signs of cognitive decline, these results suggest that ceiling effects for the women may have limited our ability to detect an association between CRF and episodic memory, if one does exist. Having a sample of women with a greater range of memory function would allow for a more powerful examination of gender specificity in the relationship between CRF and episodic memory.

### 4.3 Limitations

Due to the cross‐sectional nature of the design, causal inferences regarding the influence of CRF on the volume of the hippocampus or episodic memory cannot be made. Studies aimed at manipulating CRF (e.g., exercise training) and determining whether a change in CRF is necessary and/or sufficient to increase hippocampal volume or improve memory in at‐risk populations are needed to more fully test these hypotheses. Further, longitudinal studies similar to the study by Vidoni et al. (2012) investigating whether maintenance of CRF is beneficial for an at‐risk population are warranted. Another limitation is the restricted range in RAVLT performance in the women participants. A greater range in memory performance would allow more precise investigation of gender differences. Additionally, although the amount of time between the CRF test, MRI scan, and the RAVLT was not a significant predictor in either regression model, it is a limitation. CRF is known to decline with age, and meta‐analyses of cross‐sectional data demonstrate 7–10% decline in CRF per decade in adult men and women (Fitzgerald, Tanaka, Tran, & Seals, 1997; Wilson & Tanaka, 2000). Thus, we might expect a small (<l %) decline for the current study. This is unlikely to have a meaningful effect on the observed relationships. This was partially controlled for by adding a time interval variable into the statistical models; however, collecting CRF, hippocampal volume, and episodic memory in closer temporal proximity would improve future research. In addition, although the range of CRF values in the present investigation were consistent with previous research, it is possible that there was a limited range with insufficient numbers of low and high‐fit individuals to fully test the association between CRF and hippocampal volume in the entire sample. Although our sample size was sufficient to test our primary aims, we may have been underpowered when examining the groups stratified on gender.

### 4.4 Summary

The findings of this study add to a growing body of literature investigating whether a modifiable risk factor such as CRF may be associated with brain health in later adulthood, particularly in individuals at elevated risk for AD. Although our hypotheses were not supported for the entire sample, CRF was significantly associated with hippocampal volume in women and episodic memory in men. These results are in partial agreement with previous research showing benefits of self‐reported physical activity in those at genetic risk for AD (Boots et al., 2014; Okonkwo et al., 2014; Smith et al., 2013, 2014). However, our potential gender‐specific findings highlight the need for further research on the differential effects that gender and AD risk factors have on both brain and cognitive health. Gender differences potentially may have gone unnoticed in previous studies that used gender as a covariate, or not included gender as a variable of interest. It is important to note that these results do not preclude the possibility that a change in fitness (e.g., exercise training) may be more relevant for improving or maintaining brain health in older adults (Colcombe & Kramer, 2003; Erickson et al., 2011). Interventions designed to test whether fitness adaptations are necessary for neurobiological adaptations are needed to more fully elucidate the impact of exercise on brain health in at‐risk populations.

## Conflict of Interest

None declared.
