# Supplementary material for: Relationships between cardiorespiratory fitness, hippocampal volume, and episodic memory in a population at risk for Alzheimer's disease
Source: Brain Behav. 2017 Feb 17;7(3):e00625. doi: 10.1002/brb3.625 (PMC5346514; doi:10.1002/brb3.625)
Supplement: Supplementary file 1 [file BRB3-7-e00625-s001.docx]

Supplementary material

| Table 1: Relationship between CRF and additional RAVLT metrics | | | |
| --- | --- | --- | --- |
| **RAVLT**  **metric** | **Analytic Sample**  ***p-*value** | **Men**  ***p-*value** | **Women**  ***p-*value** |
| Proactive  Interference | .299 | .116 | .927 |
| Forgetting | .711 | .871 | .343 |
| Retroactive Interference | .380 | .149 | .872 |

Proactive interference = B1 minus A1; Forgetting = A5 minus A7; Retroactive interference = A6 minus A5

All regression models include the same covariates as outlined in the manuscript
